# The Telomeric Cdc13 Protein from Yeast Hansenula polymorpha

**DOI:** 10.32607/actanaturae.10944

**Published:** 2020

**Authors:** A. N. Malyavko, O. A. Dontsova

**Affiliations:** Faculty of Chemistry and Belozersky Institute of Physico-Chemical Biology, Lomonosov Moscow State University, Moscow, 119991 Russia; Skolkovo Institute of Science and Technology, Moscow, 121205 Russia; Shemyakin-Ovchinnikov Institute of Bioorganic Chemistry of the Russian Academy of Sciences, Moscow, 117997 Russia

**Keywords:** telomeres, telomerase, Cdc13

## Abstract

Telomeres are special structures at the ends of chromosomes that play an important role in the protection of the genetic material. Telomere composition is very diverse; noticeable differences can often be observed even among closely related species. Here, we identify the homolog of telomeric protein Cdc13 in the thermotolerant yeast *Hansenula polymorpha*. We show that it can specifically bind single-stranded telomeric DNA, as well as interact with the Stn1 protein. In addition, we have uncovered an interaction between Cdc13 and TERT (one of the core components of the telomerase complex), which suggests that Cdc13 is potentially involved in telomerase recruitment to telomeres in *H. polymorpha*.

## INTRODUCTION

Telomeres are protective caps on the tips of eukaryotic chromosomes, which consist of repeated GC-rich DNA sequences bound by specialized telomeric proteins. The specific architecture of the chromosome ends is required to prevent their recognition by the double-strand break repair system. Telomeres are dynamic structures: the length of telomeric DNA varies between chromosomes in one cell and depends on many factors regulating its shortening (incomplete end replication and degradation) or lengthening (recombination and telomerase action) [1]. The RNA–protein complex telomerase carries telomerase RNA (TER) with a short domain acting as a template for telomeric repeat synthesis, telomerase reverse transcriptase (TERT), and a number of ancillary proteins modulating the synthesis of telomeric DNA.

In most organisms, telomeres carry a short G-rich single-stranded region (the 3’-overhang) in addition to double-stranded DNA. In the budding yeast *Saccharomyces cerevisiae*, the 3’-overhang is associated with the Cdc13 protein [2, 3], which plays a key role in telomere biogenesis [4]. Thus, a Cdc13-1 mutation leads to the accumulation of single-stranded telomeric DNA and RAD9-dependent G2/M cell cycle arrest [5]. Some mutations in Cdc13 increase the length of telomeric DNA (e.g., V133E, K50Q, cdc13-5Δ894-924) [6, 7, 8], while L91R and P235S mutations cause telomere shortening [6, 9]. Strains carrying the cdc13-2E252K mutation are phenotypically identical to those where telomerase genes have been deleted [2]. These differences are related to the disruption of protein–protein interactions in which various domains of the Cdc13 protein are involved. This protein consists of four domains with an OB-fold structure. Cdc13 binds telomeric ssDNA via the OB- 3DBD domain with high affinity and specificity [10, 11]. The N-terminal OB1 domain is necessary for protein dimerization; it is also involved in the recruitment of DNA polymerase α for the synthesis of the C-rich telomere strand [6, 7, 12, 13]. The OB2 domain carries the nuclear localization signal and presumably contributes to protein dimerization and binding to other protein partners [14, 15]. Between OB1 and OB2, there lies the RD domain that exerts a favorable effect on telomeric DNA synthesis by recruiting telomerase to telomeres and activating it [16]. The RD domain consists of two short motifs that are responsible for the interactions with the telomerase accessory protein Est1 [17]. The Cterminal domain OB4 is likely involved in the interaction with Stn1 [4, 8, 14, 18]. The Cdc13, Stn1, and Ten1 proteins form the CST complex, which is regarded as a telomere-specific analog of the RPA complex and plays a crucial role in telomeres of many eukaryotic organisms [19].

The telomere sequences of the budding yeast have undergone significant modifications during evolution; nevertheless, the 3’-overhangs are bound to Cdc13 homologs in most of the studied species [20]. However, in many *Candida *species, Cdc13 homologs are duplicated (Cdc13A and Cdc13B) [21]. Furthermore, each homolog carries only two (out of four) OB-fold domains, which seem to correspond to OB3DBD and OB4 [22, 23]. Despite the loss of the OB1 and OB2 domains, these proteins can bind telomeric DNA and Stn1; they are involved in telomere length regulation and are prone to dimerization [23, 24]. In *C. parapsilopsis*, only the Cdc13A/ Cdc13B heterodimer can bind DNA with high affinity [25].

In this study, we describe the Cdc13 homolog in thermotolerant yeast *Hansenula polymorpha*, a species that is evolutionarily distant both from *S. cerevisiae* and yeast species belonging to the genus *Candida*. We have found that HpCdc13 is close to *C. albicans *Cdc13 in terms of its domain architecture; however, the genome carries a single copy of the gene encoding it. HpCdc13 shares the properties reported for Cdc13 in other yeasts: it specifically binds to telomeric ssDNA, dimerizes, and interacts with Stn1. Furthermore, we have demonstrated that although HpCdc13 lacks the RD domain, it can interact with *H. polymorpha *telomerase.

## EXPERIMENTAL

**Expression and isolation of protein HpCdc13**

The *Cdc13 *gene from the *H. polymorpha *strain DL-1 (ATCC 26012, or *Ogataea parapolymorpha *DL-1) was cloned into the pET30aTEV vector (kindly provided by Daniela Rhodes (Cambridge, MRC LMB, UK)), which codes for the 6His and S tags that are inserted into the N-terminus of the protein and can be cleaved by TEV protease. The resulting plasmid was used to transform the *Escherichia coli *BL21 Star (DE3) pRARE strain. Protein synthesis was induced using 1 mM isopropylthio- β-*D*-galactoside at 21°C for ~ 16 h.

The cells were precipitated by centrifugation, resuspended in lysis buffer (50 mM bis-Tris-propane, pH 8.0, 500 mM NaCl, 10 mM β-mercaptoethanol, 10% glycerol, 0.05% Tween 20, 30 mM imidazole, and Halt protease and phosphatase inhibitor cocktail (1×) (Thermo Fisher Scientific, USA)), and lysed ultrasonically (amplitude 80%, twice for 2 min: 3 s every 10 s). The protein was incubated for 30 min in the presence of Ni–NTA agarose, washed with the lysis buffer four times, and eluted with the lysis buffer supplemented with 300 mM imidazole.

The buffer was then replaced with a TEV buffer (50 mM bis-Tris-propane, pH 8.0, 300 mM NaCl, 1 mM dithiothreitol, 10% glycerol, 0.05% Tween 20, 0.3 mM PMSF) in PD Minitrap G-25 gel filtration columns 25 (Sigma, USA). The 6His-S tag was cleaved after incubation with recombinant TEV protease (50 μg per mg Cdc13) at +4°C for ~16 h.

The cleaved tag and TEV protease (which also contains the 6His epitope) were removed by additional purification on Ni–NTA agarose. HpCdc13 lacking the tag can bind to Ni–NTA under these conditions but is easily eluted by the TEV buffer supplemented with 50 mM imidazole. This buffer was then replaced with a storage buffer (50 mM bis-Tris-propane, pH 8.0, 300 mM NaCl, 1 mM dithiothreitol, 5% glycerol, 0.3 mM PMSF) on a PD Minitrap G-25 column (Sigma) and stored at +4°C. Protein concentration was measured spectrophotometrically according to absorbance at λ = 280 nm (the extinction coefficient was calculated using the ExPaSy ProtParam tool).

**Polyacrylamide gel electrophoresis analysis of the binding between Cdc13 and DNA**

Oligonucleotides were labelled at the 5’-end with T4 polynucleotide kinase and [γ-32P]ATP (Thermo Fisher Scientific) according to the manufacturer’s protocol and purified using Illustra MicroSpin G-25 columns (GE Healthcare). Binding was conducted in 20 μL of the mixture containing 0.1 nM oligonucleotide, 1–00 nM Cdc13, 10 mM bis-Tris-propane, pH 8.0, 100 mM NaCl, 0.5 mM dithiothreitol, 5% glycerol, and 0.5 mg/mL BSA. The mixture was incubated at room temperature for 20 min, and 1 μL of a gel-loading buffer (0.5× Tris-borate buffer, 50% glycerol, bromophenol blue, and xylene cyanol) was added. The mixture was then loaded onto an 8% polyacrylamide gel (containing 1×Tris-borate buffer and 5% glycerol). Electrophoresis was conducted for 35 min at 180 V at room temperature. The radioactive signal was detected using a Typhoon FLA 7000 system (GE Healthcare).

**Yeast two-hybrid system**

The vectors pGADT7 and pGBKT7 (to produce proteins fused to the Gal4-AD and Gal4-BD domains, respectively) and the *S. cerevisiae *strain AH109 were kindly provided by S.S. Sokolov (Laboratory of Biomembrane Photochemistry, Lomonosov Moscow State University). The analyzed *H. polymorpha *DL-1 genes were cloned into the vectors pGADT7 and pGBKT7 at the SmaI/ NdeI and SmaI/NotI sites, respectively (except for* CDC13*, which was cloned into the pGADT7 plasmid at the SmaI/EcoRI sites). The region coding for the amino acid residues 1–591 was cloned for EST1, since an attempt at expressing the full-length protein in the strain AH109 did not yield viable colonies. It is worth noting that this Est1 fragment is highly conserved: it is known that the respective fragment from *Kluyveromyces lactis* yeast contains all the domains required for the interaction with Cdc13 [17]. The analyzed pairs of plasmids were cotransformed into the strain AH109 according to the protocol described in [26]; clones were chosen using the SC-Leu-Trp selective medium. Individual colonies were resuspended in sterile water and seeded onto plates with 2% agar and the required selective medium (SC-Leu-Trp or SC-Leu-Trp-His).

## RESULTS AND DISCUSSION

**Search for an Cdc13 homolog in *H. polymorpha***

**Fig. 1:**
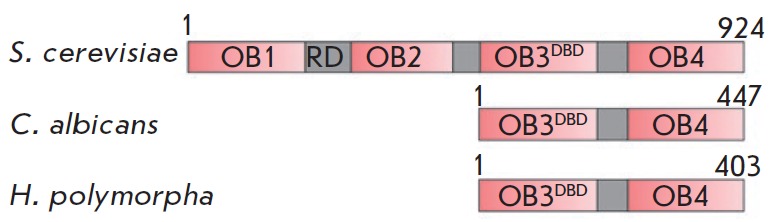
Schematic of the domain organization of Cdc13 homologs derived from the indicated organisms. The first and last amino acid residues are specified

In order to identify the Cdc13 homolog, an iterative search across the database of all annotated budding yeast proteins was performed using the PSI-BLAST tool (sequence of *C. albicans *Cdc13A were used as a search query). As a result, an open reading frame HPODL_00415 (referred to as W1QJ57 in the Uniprot database) was detected in *H. polymorpha *DL-1; its length was 403 a.a., much shorter than the ScCdc13 (924 a.a.) but approximately coinciding with the length of CaCdc13 (447 a.a.). The degree of homology between CaCdc13 and HPODL_00415 (further referred to as Hp- Cdc13) is very low: the proteins share only 15% identical and 31% similar amino acids, which is common among homologs of telomeric proteins. We also conducted a search for HPODL_00415 homologs among proteins with a known structure using the HHpred server, which performs alignments taking into account both the amino acid sequence and the predicted secondary protein structure. It turned out that the N-terminal portion of HpCdc13 is similar to the DNA-binding OB3DBD domain of *S. cerevisiae *Cdc13 (PDB: 1KXL_A). No second *CDC13 *copy in the genome of *H. polymorpha* DL-1 was detected. Thus, we conclude that we have identified a possible homolog of the Cdc13 protein in* H. polymorpha *cells, with its domain architecture probably similar to that of CaCdc13 (two OB-fold domains corresponding to OB3DBD and OB4 ScCdc13) (*Fig. 1*).

**DNA-binding properties of** *H. polymorpha* **Cdc13**

**Fig. 2:**
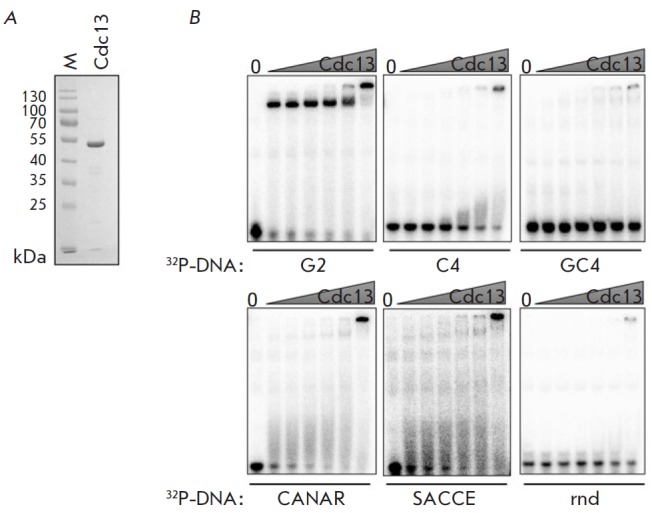
DNA binding properties of the recombinant Cdc13 from *H. polymorpha*. A – analysis of isolated and purified recombinant Cdc13 in a denaturing polyacrylamide gel. “M” – marker. B – 0.1 nM oligonucleotide was incubated with increasing concentrations of Cdc13 (1, 3, 10, 30, 100, 300 nM) and analyzed in the native polyacrylamide gel. Oligonucleotide names are specified under each electrophoregram (refer to *Table* 1 for sequences)

In order to confirm that the identified Cdc13 homolog can be a factor associated with the 3’-overhang of* H. polymorpha *telomeres, we studied its DNA-binding ability *in vitro*. We expressed HpCdc13 in *E. coli *and isolated the recombinant protein by affinity chromatography (*Fig. 2A*). The resulting protein product was incubated with various DNA oligonucleotides, and the of their binding was evaluated according to the changes in electrophoretic mobility in the native polyacrylamide gel. As one would expect, HpCdc13 binds to the G2 oligonucleotide that carries two telomeric repeats of *H. polymorpha* (*Fig. 2B*, *Table*). The observed interaction is rather strong: more than 50% of DNA oligonucleotide G2 is bound into a complex at a Cdc13 concentration as low as 1 nM (*Fig. 2B*). Under the same conditions, Cdc13 does not bind the DNA oligonucleotides C4 and GC4 carrying four telomeric C-strand repeats and four repeats of double-stranded telomeric DNA, respectively, or the nontelomeric control oligonucleotide rnd. Therefore, Cdc13 specifically interacts with the G-rich telomeric 3’-overhang. We also tested the G-rich oligonucleotides whose sequence differed from that of the telomeric DNA of *H. polymorpha* (oligonucleotides SACCE and CANAR carrying the telomeric repeats of *S. cerevisiae *and *C. arabinofermentans*, respectively; *Table*). It turned out that Cdc13 can bind to these oligonucleotides; however, the resulting complexes were unstable (as opposed to the Cdc13–G2 complex) and dissociated during electrophoresis. Hence, we have demonstrated that the identified Cdc13 homolog can recognize the 3’-overhang of* H. polymorpha *telomeres.

**Table:** The DNA oligonucleotides used in this study

Oligonucleotide	Sequence (5’ → 3’)
G2	GTAGATACGACTCACTGGGTGGCGGGGTGGCG
C4	GCCACCCCGCCACCCCGCCACCCCGCCACCCC
GC4 (sense)	GGGTGGCGGGGTGGCGGGGTGGCGGGGTGGCG
GC4 (antisense)	CGCCACCCCGCCACCCCGCCACCCCGCCACCC
CANAR	GGTGTTGGGTGTTGGGTGTTGGG
SACCE	GTGTGTGGGTGTGTGGGTGTGTG
rnd	GTAGATACGACTCACTGTAGATACGACTCACT

**HpCdc13 as a component of the CST complex**

**Fig. 3:**
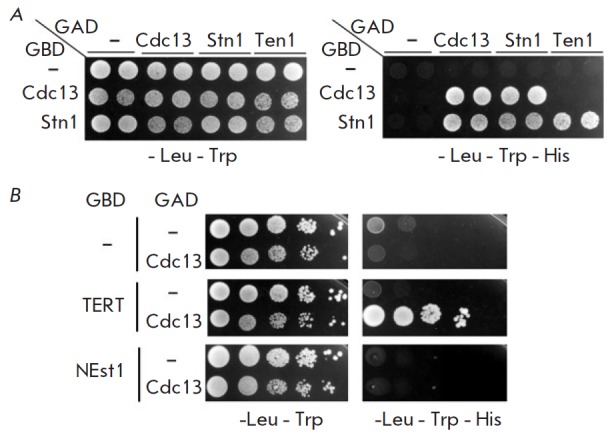
Protein–protein interactions of Cdc13 from *H. polymorpha *identified using the yeast two-hybrid system. *A *– AH109 colonies expressing pairs of the indicated proteins (fused to Gal4-BD (GBD) or Gal4-AD (GAD)) were diluted to A_600_ ~ 0.05, plated to a SC medium lacking the indicated amino acids, and incubated at 30°C for four days. Two colonies of each strain were analyzed. *B *– same as *A*, but with different protein pairs. Cultures with A_600_ ~ 0.5 and four tenfold serial dilutions were plated in this case

Another important distinctive feature of yeast Cdc13 proteins is that they are the components of the CST complex. Within this complex, Cdc13 directly comes into contact with the Stn1 protein, which in turn is bound to Ten1 [19]. We used a yeast two-hybrid system based on the *S. cerevisiae *strain AH109, with *HIS3* utilized as the reporter gene, to verify whether these interactions exist for *H. polymorpha *homologs*. *In this system, protein binding can be detected according to the ability of the strain to grow in a histidine-free medium. Indeed, we observed interactions between the* H. polymorpha *Cdc13–Stn1 and Stn1–Ten1 protein pairs but not for the Cdc13–Ten1 pair (*Fig. 3A*) (as has been reported for other yeast species). Furthermore, our yeast two-hybrid data suggests that *H. polymorpha* Cdc13, as well as Stn1, is capable of dimerizing (*Fig. 3A*), which has also been reported earlier for other yeast species [4]. Thus, *H. polymorpha *Cdc13 can be a component of the CST complex.

**The possible function of the Cdc13 protein at** *H. polymorpha* **telomeres**

A small size (namely, the absence of two N-terminal OB-fold domains and the RD domain between them) is an important feature shared by the homologs of Cdc13 from *C. albicans *and *H. polymorpha*, which differentiates them from *S. cerevisiae *Cdc13 (*Fig. 1*). In *S. cerevisiae* cells, this region is responsible for the interaction between Cdc13 and the Est1 component of the telomerase complex, which is necessary for telomerase loading onto the 3’-end of telomeres and synthesis of telomeric DNA [17]. Does the absence of the RD domain in the truncated Cdc13 homologs mean that they have lost this important function? We have tested HpCdc13 and HpEst1 using the two-hybrid system and observed no interaction between these proteins (*Fig. 3B*), which is consistent with a lack of the RD domain in *H. polymorpha* Cdc13. However, we observed an interaction between HpCdc13 and HpTERT – the key component of the telomerase complex. This result indicates that *H. polymorpha *Cdc13 can still recruit telomerase to telomeres through a mechanism differing from that in* S. cerevisiae*.

## CONCLUSIONS

In this study, we have identified a *H. polymorpha* protein that can act as a factor associated with the telomeric 3’-overhang. Similar to Cdc13 from *Candida* yeasts, the detected protein differs significantly from its *S. cerevisiae *homolog in terms of its structure. Our findings provide evidence in favor of an interaction between HpCdc13 and the telomerase catalytic subunit, which probably is important for association between telomerase and the telomeric 3’-overhang. These data offer additional insight into the mechanisms of telomere length regulation in eukaryotes.

## Abbreviations

BSA: bovine serum albumin
ssDNA: single-stranded DNA
PMSF: phenylmethanesulfonyl fluoride
